# Genetic Susceptibility and Protein Expression of Extracellular Matrix Turnover-Related Genes in Oral Submucous Fibrosis

**DOI:** 10.3390/ijms21218104

**Published:** 2020-10-30

**Authors:** Ru-Hsiu Cheng, Yi-Ping Wang, Julia Yu-Fong Chang, Yu-Hwa Pan, Mei-Chi Chang, Jiiang-Huei Jeng

**Affiliations:** 1 Department of Dentistry, Chang Gung Memorial Hospital, Taipei 105, Taiwan; life86778231@yahoo.com.tw (R.-H.C.); shalom.dc@msa.hinet.net (Y.-H.P.); 2 College of Oral Medicine, Taipei Medical University, Taipei 110, Taiwan; 3 School of Dentistry, National Taiwan University Medical College, Taipei 100, Taiwan; ypwang0530@ntu.edu.tw (Y.-P.W.); jyfchang@ntu.edu.tw (J.Y.-F.C.); 4 Department of Dentistry, National Taiwan University Hospital, Taipei 100, Taiwan; 5 Biomedical Science Team, Department of Nursing, Chang Gung University of Science and Technology, Taoyuan 333, Taiwan; 6 College of Dental Medicine, Kaohsiung Medical University, Kaohsiung 807, Taiwan; 7 Department of Dentistry, Kaohsiung Medical University Hospital, Kaohsiung 807, Taiwan

**Keywords:** areca nut, betel quid, extracellular matrix turnover, genetic susceptibility, oral cancer, submucous fibrosis

## Abstract

Betel quid (BQ) chewing increased the risk of oral cancer and oral submucous fibrosis (OSMF), an oral premalignant disorder (OPMD) with malignant transformation potential. BQ components such as areca nut (AN), trauma by coarse AN fiber, catechin, copper, alkaloids, stimulated reactive oxygen species (ROS), inflammation and cytotoxicity are suggested to be the contributing factors. They may induce tissue inflammation, proliferation of fibroblasts and collagen deposition, myofibroblast differentiation and contraction, collagen cross-links and inhibit collagen phagocytosis, finally leading to the development of OSMF and oral cancer. These events are mediated by BQ components-induced changes of extracellular matrix (ECM) turnover via regulation of TGF-β1, plasminogen activator inhibitor-1 (PAI-1), cystatin, lysyl oxidase (LOX) and tissue inhibitors of metalloproteinases (TIMPs) and metalloproteinases (MMPs). Genetic susceptibility is also involved in these disease processes. Further understanding the molecular mechanisms of BQ-induced OSMF and oral cancer can be helpful for future disease prevention and treatment.

## 1. Introduction

### 1.1. Epidemiology and Risk Factor of Oral Submucous Fibrosis (OSMF)

OSMF is defined as an insidious chronic disease affecting any part of the oral cavity and sometimes pharynx. Clinically OSMF presents with burning and pain of the mouth, oral mucosal atrophy with fibrosis of submucosal tissues, mucosal rigidity and reduction in mouth opening. OSMF is common in India, Sri Lanka, Taiwan and other southeast Asian countries, and has started spreading to Europe and North America [1,2]. In Taiwan, the prevalence of OSMF increased from 8.3/100,000 person in 1996 to 16.2/100,000 person in 2013 [3]. The prevalence of betel quid (BQ) chewing, tobacco smoking and alcohol drinking habits in patients with oral premalignant lesions are about 82.9%, 95% and 22.7%, respectively [4]. OSMF may occur at any age but is frequently seen at the age of 21–30 years old. Male to female ratio is around 11:1 [5], possibly due to intrinsic differences between genders with/without the BQ chewing habit. Middle-age chewers are the more commonly involved population in India [6]. BQ components are suggested to be the major etiologic factors for OSMF and oral squamous cell carcinoma (OSCC), due to their content of inflammatory, genotoxic, carcinogenic and fibrogenic factors such as areca nut (AN), lime, arecoline, catechin, catechol, copper, and reactive oxygen species (ROS) [1,7,8,9].

OSMF is an oral potentially malignant disorder (OPMD) carrying risk for malignant transformation. The malignant transformation rate of OSMF is reported to be about 5.7% after 80.9 months of follow-up [4] or 7–13% [10]. In a total of 1774 cases of OSMF and OSCC in Pakistan, 765 (43.12%) cases were OSMF alone, 472 (26.60%) cases were shown to have OSCC with malignant transformation from OSMF, whereas 537 (30.27%) cases had OSCC without OSMF [11]. A 6.8-year follow-up study also elucidated that alcohol consumption is associated with the malignant transformation of patients with oral precancer [12]. Other factors including BQ chewing habit, smoking habit, environmental heavy metal exposure [13], gender, site of lesion and histological features such as epithelial dysplasia, loss of heterozygosity, aneuploidy of DNA and human papillomavirus (HPV) infection are suggested to stimulate the progression and malignant transformation of OSMF [14].

### 1.2. BQ and OSMF—Etiology, Clinical and Histologic Features

**Figure 1:**
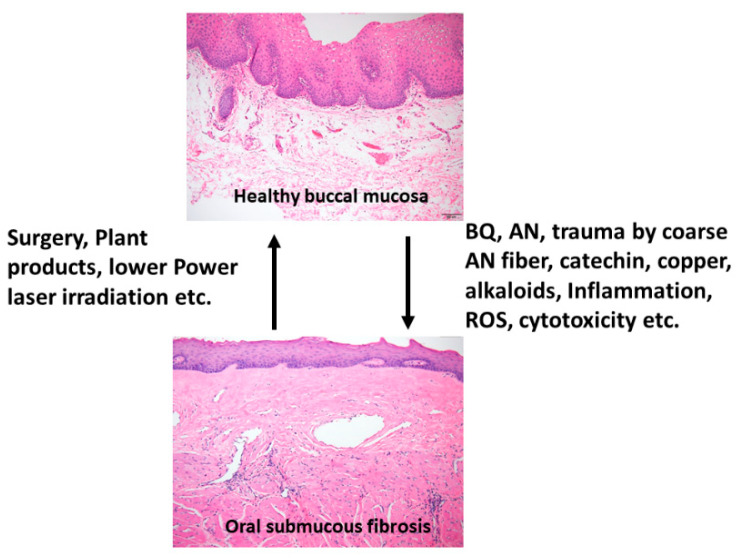
Proposed contributing factors (betel quid [BQ], areca nut [AN], coarse fiber of AN, arecoline, catechin, catechol, reactive oxygen species [ROS], inflammation etc.) for oral submucous fibrosis (OSMF) and the treatment methods (surgery, natural products, low power laser irradiation, enzymes, corticosteroid, vasodilator, antioxidants) for OSMF. The histologic pictures of (**upper panel**) normal buccal mucosa and (**lower panel**) late stage of OSMF. (H&E stain, A & B: 100×). Normal buccal mucosa is covered by nonkeratinized squamous epithelium with few short rete ridges and supported by lamina propria, which is composed of mainly loose fibrous connective tissue with some collagen fibrils dispersed. In contrast to normal mucosa, the mucosa in late stage OSMF patients shows thin parakeratinized squamous epithelium with atrophic change and a flat junction between epithelium and connective tissue. Dense collagen fiber deposition to hyalinized fibrous tissue involving lamina propria and superficial submucosa is evident. Some chronic inflammatory cell infiltration is frequently seen.

**Figure 2:**
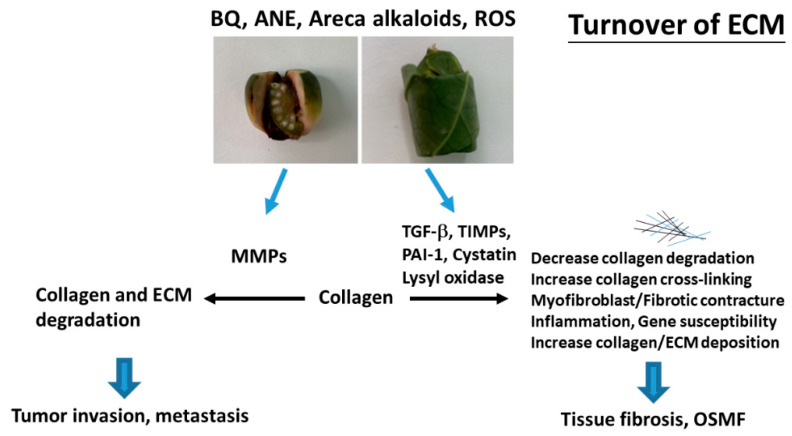
Molecular mechanisms of changes in turnover of extracellular matrix (ECM) of OSMF and tumor invasion. Collagen and ECM deposition are stimulated by transforming growth factor-β1 (TGF-β1), tissue inhibitors of metalloproteinases (TIMPs), lysyl oxidase, cystatin, and plasminogen activator inhibitor-1 (PAI-1). These events may contribute to decreased ECM degradation, increased collagen cross-linking/stability and increased collagen/ECM deposition, leading to tissue fibrosis and oral submucous fibrosis (OSMF). ECM degradation is mediated mainly via metalloproteinases (MMPs) and contributes to tumor invasion/metastasis.

OSMF is a chronic, progressive, high-risk precancerous disease characterized by juxtaepithelial inflammatory reaction, fibrosis of lamina propria, thin parakeratinized squamous epithelium with atrophic change and loss of rete peg. Increased dense collagen fiber deposition in the lamina propria occur through time and, in the end stage, dense hyalinized fibrous tissue occupies the lamina propria and even superficial submucosa, and thus results in varied degrees of mucosal rigidity (Figure 1). OSMF is accompanied by fibroelastic hyperplasia, with/without epithelial hyperplasia/dysplasia over the oral cavity or oropharynx [15,16]. Repeated trauma causes inflammation and aggravates the fibrosis while increased collagen fiber deposition, decreased amount of blood vessels and atrophic change of the epithelium occur. Progression of OSMF, thereby, may lead to loss of tissue mobility, trismus and limited mouth opening [15,16]. However, current treatment strategies are not so effective for attenuation of OSMF. BQ chewing is shown as the major etiologic factor of OSMF [1,2,17]. There are about 200–600 million BQ chewers in the world [1]. The ingredients of BQ vary in different countries. In Taiwan and Papua New Guinea, BQ comprised of AN, and slaked lime with/without inclusion of *Piper betle* inflorescence or *Piper betle* leaf (betel leaf) [1,7]. However, in India and Sri Lanka, tobacco is popularly added as one major component of BQ [1]. Among the BQ ingredients, AN components are considered to be the main causative factors in the disease process of OSMF. This is because OSMF is widespread in Taiwan where tobacco is not added into BQ. The roles of lime, betel leaf and other ingredients in the pathogenesis of OSMF await further clarification. AN contains mainly alkaloids (such as arecoline, arecaidine, guvacoline, and guvacine), catechol, catechin, transition metals (Copper, Iron) and fibers [1,7]. The contributory role of AN components to the pathogenesis of OSMF is closely associated with the induction of ROS production [18], chronic mucositis, ulcers caused by mechanical trauma from coarse AN fibers [19,20], activation of the coagulation system [21], cytotoxicity to oral epithelial cells [8], stimulation of fibroblast proliferation/contraction [22,23], collagen synthesis/deposition [22,24], myofibroblast differentiation [10], tissue inflammation [9,25] and the inhibition of collagen degradation and phagocytosis [26,27].These AN components include AN extract (ANE), areca alkaloids (arecoline, arecaidine, guvacoline, guvacine), catechin, catechol and copper (Figure 1 and Figure 2). However, only about 1% to 2% of BQ chewers develop OSMF, suggesting the presence of some predisposition factors toward mild or severe OSMF in these affected patients [17].

## 2. Genetic Susceptibility and Expression in Tissue/Organ Fibrosis

A number of studies have found the association of genetic susceptibility with tissue/organ fibrosis such as pulmonary fibrosis, systemic sclerosis, liver and kidney fibrosis [28,29,30,31]. While exogenous factors such as viral hepatitis and alcohol abuse are the common causative factors of liver fibrosis, genetic predisposition may contribute to the progression of fibrosis, cirrhosis, liver failure or hepatic carcinoma [28]. Environmental factors are the key etiologic factors of lung fibrosis, but genetic factors in host defense, aging/senescence and cell-cell adhesion may also increase the risk of pulmonary fibrosis, subsequent disease progression and poor prognosis [30]. Systemic sclerosis as an autoimmune disease may involve vascular abnormalities, immune alterations and fibrosis of skin and other internal organs, where tissue inflammation and genetic susceptibility are present [31].

Several factors including nutritional deficiency, vitamin deficiencies and hypersensitivity to various dietary constituents, may also play a part in the pathogenesis of OSMF. Epidemiological studies strongly indicate that AN is the major etiologic agent that releases alkaloids to promote fibroblastic proliferation and increase collagen formation. However, only a small population of BQ chewers develop the disease, indicating that difference in genetic susceptibility plays a role in this event [32]. About 7–12% of OSMF cases progress into oral malignancy [33]. Additionally, a few individuals developed the disease after only a few contacts with BQ [34]. Therefore, the relationship between gene and OSMF is still uncertain and awaits clarification.

## 3. BQ and Collagen Turnover

### 3.1. Collagen-Related Genes

The extracellular matrix (ECM) provides a three-dimensional scaffold for cells via connection of cell surface receptors with various ECM components such as collagen, fibronectin, elastin and nonfibrillar proteins including proteoglycan, hyaluronan and glycoproteins [35]. Hypoxia and collagen-rich conditions also intensify cancer progression [36]. Impairment of ECM and ECM-cell interaction play important roles in various diseases such as osteoarthritis, fibrosis, cancer and genetic diseases [35]. OSMF is such a collagen-related disorder, with dense collagen deposition in the oral submucosa as its main characteristic feature. It has been found that fibroblasts from buccal mucosa exposed to areca alkaloid, due to BQ chewing, may result in the accumulation of collagen [22]. Therefore, it is hypothesized that collagen-related genes might play a role in OSMF pathogenesis. Transforming growth factor-β1 (TGF-β1), lysyl oxidase (LOX), cystatin (CST3), plasminogen activator inhibitor-1 (PAI-1), matrix metalloproteinases (MMPs) and tissue inhibitors of metalloproteinases (TIMPs) proved to be involved in the turnover of ECM, wound healing, tumor invasion and metastasis (Figure 2) [37,38]. However, few studies have compared protein expression and polymorphisms of the collagen-related genes situated on different chromosomes between OSMF patients and healthy controls. Some noninvasive markers (serum markers, urinary markers and image tissue stiffness markers) have been developed for evaluation of organ fibrosis, but still with some limitations [39]. Chiu et al. (2002) suggested that polymorphisms of collagen-related-genes can serve as markers of disease susceptibility in patients with OSMF [40]. Further studies on the development of noninvasive early disease markers of OSMF are crucial for disease prevention and treatment in the future.

### 3.2. Role of Collagen 1A1 and Collagen 1A2 (COL1A1 and COL1A2)

Collagen in OSMF has proved to be histologically, biochemically and immunochemically normal, but increased in amount. Initially, deposition of type I and type III collagen in OSMF is similar to that of normal oral mucosa. However, type III collagen is gradually replaced by type I collagen as the disease progresses, eventually leading to a collagen I predominant microenvironment [41]. In the early and intermediate stages of OSMF, increased tenascin, perlecan, fibronectin, type III collagen and elastin are found in the lamina propria of oral mucosa, the submucosal layer and around muscle [42]. In advanced OSMF, these decrease and are replaced entirely by type I collagen [42], suggesting changes of ECM during disease progression. Reichart et al. (1994) found the loss of type III collagen, type VI collagen and tenascin in the fibrotic zone of OSMF tissues [43]. Concomitant over-expression of type I collagen and colligin/Hsp47, a stress protein and molecular chaperon for collagen, in OSMF tissues was also observed [44].

Interestingly, copper levels in serum/plasma/saliva, buccal tissue and cytological smear of oral mucosa cells are elevated in OSMF patients compared to control subjects [45,46,47,48]. Salivary copper levels showed positive association with the histological grade of OSMF [49]. Copper is a cofactor for LOX that is important for collagen and elastin cross-linking/maturation as well as insolubility, and maintaining the rigidity and structural integrity of ECM [50]. Its salivary level increases rapidly after BQ chewing [51]. Copper has been shown to stimulate collagen synthesis, but not proliferation of oral fibroblasts [24], suggesting the involvement of copper in mediating OSMF. Tetrathiomolybdate, a copper chelator, is thus considered to have a therapeutic effect on fibrotic diseases by repressing LOX expression [52], and possibly also for the prevention/treatment of OSMF and OSCC. Kuo et al. reported the presence of more type I collagen mRNA and type I collagen trimers in OSMF fibroblasts relative to normal buccal fibroblasts [53]. Furthermore, polyphenol and catechin fractions of AN may induce collagen cross-linking [26]. In addition, ANE and arecoline were shown to stimulate collagen production in fibroblasts in vitro [54,55].

The genotypes of type I collagen show association with the highest risk of OSMF [40]. Type I collagen is processed from type I procollagen, a triple-stranded, rope-like molecule combining two COL1A1 gene-encoded alpha1 chains and one COL1A2 gene-encoded alpha2 chain. Comparing the frequency distribution of CC, CT, TT on the COL1A1 gene in chromosome 17q, the high OSMF risk allele seems to be *CC* in the low-exposure group, while TT in high-exposure group. Comparing the frequency distribution of AA, AB, BB on the COL1A2 gene in chromosome 7q further elucidated that the high OSMF risk allele seems to be AA in the low-exposure group, while BB in high-exposure group [40].

## 4. BQ and MMPs

MMPs are a family of neutral proteases that degrade ECM produced by a variety of cells [56]. Currently, 28 human MMPs have been identified, and these enzymes are classified according to their substrate specificity and structural similarities. The major subgroups are collagenases (MMP-1), gelatinases (MMP-2 and MMP-9), stromelysin (MMP-3) and membrane-bound MMPs. One major question is whether inhibition of fibrogenesis and induction of fibrinolysis may resolve tissue fibrosis and thus relieve OSMF [57]. In addition to regulating ECM proteolysis, MMPs may also process a number of biologically active proteins such as cytokine, chemokines, cell-surface proteins, TGF-β1 and other inflammation-related molecules [56], which contribute to tissue fibrosis. Moreover, MMPs expression is involved in tumor invasion and metastasis of OSCC.

Various types of MMPs are expressed and activated in patients with OSMF as well as head and neck squamous cell carcinoma (HNSCC) [58]. Gene polymorphisms are suspected to influence the gene transcription and expression level in OPMD and malignant lesions.

A number of studies aimed to explore the association of protein expression and single nucleotide polymorphisms (SNP) of MMP-1, MMP-2 (−1306 C/T), MMP-3 (−1171 5A > 6A), and MMP-9 (−1562 C/T) promoter in OSMF and HNSCC. Cases will be discussed below.

### 4.1. Collagenase-1 (COLase, MMP-1)

Collagenase-1, also called MMP-1 or interstitial collagenase, is one of the major subgroups of the MMP family and the principal human enzyme that cleaves collagens, including type I, II, III, VII and X. It is shown to be important for photoaging and photocarcinogenesis [59]. It is produced by a wide variety of cells such as stromal fibroblasts, macrophages, endothelial cells, epithelial cells and tumor cells [60]. In addition, it is secreted as a latent precursor known as procollagenase and can be activated by many pathways [61]. MMP-1 activity has been found to be lower in OSMF than in normal oral mucosa, varying the collagen metabolism of the patients. But there is no statistically significant difference between different histological grades of OSMF [62,63]. On the contrary, an elevated expression of MMP-1 in OSMF (*n* = 30) is found in comparison with normal mucosa (*n* = 10), but no difference is noted between different histological grades of OSMF [63]. Rajendran et al. (2006) found elevated expression of MMP-1 in stromal cells of OSMF [64]. Lee et al. (2008) also found elevated expression of MMP-1 in OSCC patients with a BQ chewing habit [65]. A higher expression of MMP-1 is also discerned in survived OSCC cells after exposure to BQ components and arecoline.

The MMP-1 promoter region (−1607 1G/2G) has been found to affect its transcriptional activity and may contribute to carcinogenesis and metastasis of cancers. Chaudhary et al. (2011) found that SNPs in the MMP-1 promoter region (−1607 1G/2G) are associated with the susceptibility of BQ chewers to OSMF and HNSCC in India. Habitual BQ chewing and alcohol consumption may enhance the expression of the 2G allele of MMP-1 genes in OSMF and HNSCC patients [58]. Similarly, the 2G genotype of the MMP-1 promoter is observed in higher frequency in both OSCC and OSMF patients, compare to controls. However, there is also a study indicating the MMP-1 promoter region (−1607 1G/2G) polymorphism increases the risk of OSCC, but not OSMF [66].

### 4.2. MMP-2 and MMP-9 (Gelatinase-A and Gelatinase-B)

Both MMP-2 (Gelatinase-A) and MMP-9 (Gelatinase-B) are Zn^2+^-dependent endopeptidases with similar structures. However, their physiological distributions show some differences. MMP-2 is expressed by a wide variety of cell types in normal conditions, while MMP-9 is expressed in only few cell types including trophoblasts, osteoclasts, leukocytes, dendritic cells and their precursors [67]. MMP-2 primarily degrades proteins in the ECM and basement membrane as its primary function. It also degrades type I, IV, V, VII and X collagens, laminin, elastin, fibronectin and proteoglycans [68].

The MMP-2 gene is located on chromosome 16q. Price et al. reported that C to T substitution at −1306 in the promoter region of MMP-2, disrupts the Sp1-binding site and results in reduction of its transcriptional activity. On the contrary, the −1306C allele may enhance its transcription level [69]. Therefore, individuals who carry the CC genotype express higher MMP-2 protein than those who carry the TT or CT genotype. Lin et al. (2004) assessed the association of MMP-2 genotype with the risk of OSMF and OSCC by comparing 58 OSMF cases, 121 OSCC cases and 147 controls. Their data suggested no significant association between subjects carrying the *CC* genotype and the development of OSMF, but the results showed that subjects carrying the *CC* genotype had a two-fold increased risk for developing OSCC when compared with the CT or TT genotypes [70].

MMP-9 proteolytically degrades ECM components such as decorin, elastin, fibrillin, laminin, gelatin and type IV, V, XI and XVI collagen. Moreover, it activates growth factors like proTGF-β and proTNF-α [71]. Expressions of MMP-2, MMP-9, TIMP-1 and TIMP-2 in OSMF are found to be higher than in healthy oral mucosa [64]. A systemic review for analysis of MMP-9 expression in 182 OSCC patients, 432 patients with OPMDs (146 lichen planus, 264 oral leukoplakia, and 20 OSMF) relative to healthy control tissues (352 patients) has been done. It showed that MMP-9 levels were elevated in mucosa tissues, saliva and serum of patients with OPMDs compared to controls [72]. This over-expression of various MMPs also conforms to the natural tendency of oral mucosa to regenerate upon chronic tissue damage by switching off the fibrogenic effect [57]. Increased levels of various MMPs in serum have been shown to be valuable biomarkers in liver cirrhosis and other fibrotic diseases [57]. Arecoline may stimulate TIMP-1 expression but inhibit MMP-2 and MMP-9 expression of buccal mucosal fibroblasts [73]. Interestingly, oral keratinocytes and SAS tongue cancer cells express MMP-9, and ANE stimulates MMP-9 but suppresses TIMP-1 and TIMP-2 secretion via differential signaling pathways [74,75]. Increased expressions of MMP-2, MMP-3, MMP-9, MMP-10 and MMP-13 immediately after acute liver injury prior to fibrogenesis has been reported and contributes to hepatocyte necrosis, suggesting that MMPs participate in the acute phase of hepatic injury and the activation of tissue fibrosis [76]. The concomitant synthesis and degradation of ECM is a hallmark of the dynamic nature of liver cirrhosis [76]. These results indicate that expression of MMPs can vary in different stages of OSMF and OSCC. The stimulation of MMPs by BQ components also contributes to inflammation, epithelial cell necrosis, oral mucosa atrophy and activates fibrogenesis in OSMF tissues.

The MMP-9 gene is located on chromosome 20q. The C to T transition at −1562 in the promoter region of MMP-9 leads to differential transcription and is associated with increased susceptibility to various diseases. In 2011, Chaudhary et al. (2011) explored the association of MMP-2 and MMP-9 promoter polymorphisms in OSMF and HNSCC by analysis of 1260 subjects (412 OSMF, 422 HNSCC and 426 controls). The SNPs were genotyped by polymerase chain reaction-restriction fragment length polymorphism (PCR-RFLP). The results revealed that no significant difference in MMP-2 (−1306 C/T) and MMP-9 (−1562C/T) polymorphism occurred in OSMF patients compared to healthy controls, whereas the T allele showed a significant association with increasing progression of clinicopathological grading in HNSCC [58]. In Taiwan, Tu et al. (2007) studied the MMP-9 SNPs in BQ-related OSCC (*n* = 192), OSMF (*n* = 73), and nondiseased BQ chewers (*n* = 191). They found that functional association of MMP-9 -1562 C/T polymorphism with increased risk of OSCC was only found in young BQ chewers, but not in elder populations [77]. The MMP-9 genotype also showed no association with OSMF and the lesion sites, metastasis stage and survival of OSCC patients [77].

### 4.3. MMP-3 (Stromelysin-1)

MMP-3 is able to degrade components in the basal membrane and collagens type II, V, IX and X. It can also induce the activation of other MMPs such as MMP-1 and MMP-9. The MMP-3 gene is located near chromosome 11q, and the insertion or deletion of a single adenosine at position-1171 in the promoter region of MMP-3 gene could result in different transcriptional activities. Ye et al. (2000) reported that the 6A allele has a lower promoter activity than the 5A allele in vitro [78].

Tu et al. (2006) aimed to assess the association of the MMP-3 genotype with the risk of OSCC and OSMF by analysis of 150 OSCC cases, 71 OSMF cases and 98 controls. The data showed that the frequency of the 5A genotype in the MMP-3 promoter was higher in the OSMF group than in the control group, while no significant differences were noted between the OSCC and control groups. The results indicated that the 5A genotype in MMP-3 promoter is associated with the risk of OSMF but not OSCC [79]. Simultaneous MMP-3 and MMP-9 polymorphisms also showed no marked effect on the risk or prognosis of OSCC [77].

Chaudhary et al. (2010) investigated the genotype of MMP-3 SNP by PCR-RFLP analysis in a case control study consisting of 362 participants (101 OSMF, 135 HNSCC and 126 controls). Analysis of MMP-3 polymorphism revealed the frequency of the 5A allele in OSMF, HNSCC and controls to be 0.15, 0.13 and 0.07, respectively. The difference of 5A genotype frequency between OSMF and controls was statistically significant and similar to that between HNSCC and controls. In this study, the 5A genotype had a greater than two-fold increased risk for developing OSMF, as in HNSCC compared to controls, but this phenomenon was only observed in patients less than 45 years of age. These results imply that expression of the MMP-3 genotype is associated with the 5A alleles and may play an important role in the susceptibility to develop OSMF and HNSCC [80].

Zade et al. (2017) evaluated the genotype of MMP-3 SNP by PCR analysis in a case control study consisting of 20 individuals (five OSMF, five OSCC, five normal individuals with tobacco and AN habits, and five without the habit). Their data showed that the frequency of the 5A genotype in the MMP-3 promoter was higher in the OSMF group than in the control group., but no significant difference was noted between the OSCC and control groups. The results are similar to Tu’s study. They indicated that the 5A genotype in the MMP-3 promoter is associated with the risk of OSMF, but not OSCC, in an Indian population [81]. Above all, MMP-2-1306 C to T transition is associated with high transcriptional activity of the MMP-2 gene. In MMP-3-1171 5A > 6A, the insertion or deletion of a single adenosine could alter the transcription level of the MMP-3 gene. For MMP-3, the frequency of the 5A genotype in the MMP-3 promoter region was higher in OSMF group than in the control group and had a greater than two-fold risk for developing OSMF compared to controls. However, the 5A/5A carrier alleles showed an association only in patients less than 45 years of age. Further studies with larger sample sizes are warranted.

## 5. BQ and TGF-β

The synthesis of collagens is influenced by a wide variety of mediators, including growth factors, hormones and cytokines. Among these mediators, TGF-β1 is a prominent one, which controls the proliferation, differentiation and other functions in many cell types. It also stimulates the production of collagens through regulation of intramembrane proteolysis (RIP) and activation of CREB3L1 [82]. The activation of TGF-β/Smad2 and Smad4 signaling has been found in keratinocytes and myofibroblasts in OSMF tissues [10]. Kale et al. found more adipose tissues and TGF-β1 expression in early stage OSMF tissues in India [83]. Over-expression of both TGF-β1 and TGF-β2 was reported in OSMF tissues, with a higher expression of TGF-β1 than TGF-β2. TGF-β1 is expressed mainly in epithelial cells, perivascular cells, infiltrated inflammatory cells, fibroblasts and muscle cells. The signal of TGF-β2 was mainly localized in the submucosal area with minimal involvement of the epithelium [84,85]. The mRNA level of TGF-β1 was higher in the early and middle stages of OSMF tissues than that in healthy counterparts in patients of Hunan, China [86]. An increased expression of CD105, a TGF-β1 receptor, was associated with hypoxia-induced neoangiogenic activity in OSMF, and this feature was linked to transformation from normal oral mucosa to mild and severe epithelial dysplasia [87,88,89]. Its expression was also associated with differentiation status, TNM stage, metastasis, and three-year survival rate of OSCC patients with OSMF [90].

A study in Sri Lanka showed that the difference in TGF-β1 expression was not evident among OSMF tissues in pan masala (a chewing tobacco and AN product in India) chewers without OSMF and healthy oral mucosa. Secretion of TGF-β1 in cultured fibroblasts harvested from the aforementioned specimens also showed no marked difference [91], but recent real-time PCR and immunohistochemical staining studies found the markedly elevated expression of TGF-β1, p-Smad2, connective tissue growth factor (CTGF), MMP-3 and decreased expression of bone morphogenetic protein-7 (BMP-7) in OSMF. They also showed the possible stimulatory effect of areca components (ANE, arecoline and others) on epithelial-mesenchymal transition and the expression of TGF-β1, p-Smad2, smooth muscle actin, CTGF and LIM domain kinase 1 (LIMK1) in epithelial cells [92,93,94]. Local injection of ANE and pan masala extract to buccal mucosa of Sprague Dawley rats on alternate days for 48 weeks induced OSMF-like changes with a concomitant elevated expression of TGF-β1 [95], suggesting the contribution of AN and pan masala components. Polyphenol, tannin, catechin and areca alkaloids of AN components are able to stimulate TGF-β2 and p-Smad in keratinocytes, but not gingival fibroblasts [84,93]. We also found that ANE stimulates TGF-β1 and Smad2 signaling in oral keratinocytes and SAS oral cancer cells [75], implicating the involvement of AN in the pathogenesis of OSMF. Accordingly, smad2 over-expression is reported in OSMF tissues relative to healthy tissues [96]. Curcumin may attenuate the increased expression of TGF-β1, inducible nitric oxide synthase (iNOS) and p53 in OSMF [97]. Unexpected expression of Smad 7, an inhibitor of TGF-β, is elevated in OSMF and OSCC tissues relative to normal tissues [98], and has been suggested as a promoter and diagnostic marker. Interestingly, for treatment of OSMF, glabridin as an isoflavone is shown to attenuate the arecoline-induced TGF-β1, p-smad2, collagen and fibroblast contraction [99], implicating its potential use for prevention and treatment of OSMF. However, initial attempts for systemic inhibition of TGF-β1 are disappointing because of an increase in generalized tissue/organ inflammation [100].

The association of genotypes of TGF-β1 with the risk of OSMF has been studied. Comparing the frequencies of TT, TC, CC alleles on the TGF-β1 gene on chromosome 19q, high OSMF risk seems to be associated with CC alleles in both low and high exposure groups [40]. Another study in India also found an evidently different frequency of a C to T transition (rs13306708) in the 5′UTR region of TGF-β1 between OSMF patients (68.7% C and 31.2% T [27CC, 15CT, 8TT] and control subjects (89.5% C and 10.4% T [42CC, 6CT, 2TT]), but the authors did not find any differences of polymorphisms in the promoter region and exon 1 of TGF-β1 between OSMF patients and control subjects [101]. Hsu et al. (2014) compared the TGF-β1 codon 10 T/C and codon 25 G/C polymorphisms in patients with oral premalignant lesions (*n* = 42) and healthy control (*n* = 128). They found an association of TGF-β1 codon 10 and 25 polymorphisms with the development of OPMD [102].

## 6. BQ and Lysyl Oxidase (LOX)

Lysyl oxidase (LOX), also known as protein-lysine 6-oxidase, is a copper-dependent extracellular enzyme that functions in crosslinking of collagens and elastin through posttranslational oxidative deamination of peptidyl lysine residues in their precursors. This modification stabilizes the collagen fibrillar array [103,104], and contributes to ECM stiffness and mechanical property. LOX and LOX-like proteins are involved in atherosclerosis, tissue fibrosis, tumorigenesis and metastasis through changes in protein expression and regulation of signal transduction [103]. LOX overexpression may affect the tumor microenvironment, tumor desmoplasia (fibrosis), and also stimulate anchorage-independent growth of OSCC cells [103,105,106].

LOX mRNA expression in blood cells from OSMF (*n* = 127) and control patients (*n* = 127) has been analyzed. LOX expression in blood cells from patients of OSMF can be similar (*n* = 89), lower (*n* = 11) or higher (*n* = 27) than age and sex-matched controls [107], suggesting that changes of LOX in circulating blood cells of OSMF are not evident. The activity of LOX is found to be elevated in fibroblasts cultured from OSMF patients relative to fibroblasts cultured from normal oral mucosa [108]. An epidemiological study showed elevated LOX expression in OSCC tissues relative to adjacent oral mucosa. Similarly, over-expression of LOX was also found in OSMF tissues [105]. This can be partly explained by stimulation of LOX expression in oral keratinocytes by ANE [105]. The presence of copper in BQ is found to stimulate LOX expression of fibroblasts, and thus increases collagen cross-linking and resistance to degradation [109].

LOX is encoded by the LOX gene on chromosome 5q, and defects in this gene have been linked with predisposition to thoracic aortic aneurysms and OSMF. The frequencies of three genotypic variants (AA, AG, and GG) of LOX genes in patients of OSMF and controls were evaluated. The high OSMF risk allele seems to be AA in the low-exposure group, while GG is more prevalent in the high-exposure group [40]. The differences of Arg158Gln SNPs of the LOX genotype between elder BQ chewers (control, 216 patients, without OSMF) and OSMF patients (83 patients with OSMF) through PCR-RFLP and direct sequencing were found [110]. The Arg158Gln SNP was found to be associated with earlier clinical stage of OSCC [105]. Thorawat et al. studied the LOX G473A SNP in OSMF, BQ chewers without OSMF and healthy control (*n* = 20 in each group). They did not find any LOX G473A SNPs in this Indian population [111]. From the above reports and others, LOX and LOX-like proteins are suggested to be potential therapeutic targets not only for fibrotic diseases of systemic organs [106], but also the treatment of OSMF and OSCC in the future.

## 7. BQ and Cystatin C (CST3)

In addition to LOX, cystatin C (CST3) is another molecule responsible for prevention of ECM degradation. The terminal regions of each collagen molecule are composed of terminal peptides, which function in cross-linking and enhance the strength of collagen fibers. These areas are resistant to attacks by collagenases but are susceptible to other serine and cysteine proteinases [112]. These groups of enzymes belong to the cystatin superfamily, namely the type 1 cystatins (stefins A, B), type 2 cystatins and the kininogens. Cystatin C is one of the type 2 cystatins, a class of cysteine proteinase inhibitors found in a variety of human body fluids and secretions. The major functions of this enzyme are thought to provide protection and stabilization of the collagen fibrils.

Cystatin was shown to play crucial roles in fibrosis and carcinogenesis of various organs such as lung, kidney and liver [113,114,115]. Cystatin C expression is significantly higher in OSMF tissues from patients than in normal oral mucosa. It is mainly expressed by fibroblasts, endothelial cells and inflammatory cells. Fibroblasts from OSMF were shown to have higher cystatin expression than normal fibroblasts. In addition, arecoline was found to promote cystatin C mRNA and protein expression in a dose-dependent manner [116]. An elevated cystatin M was shown to promote metastasis of OSCC by blocking cathepsin B activity and rescue tumor cells from TNF-α-induced apoptosis [117]. Salivary cystatin B level was also found to be a valuable prognostic marker for OSCC patients [118]. More studies are needed to clarify the role of various cystatins in OSMF and OSCC.

Cystatin C is encoded by the CST3 gene on chromosome 20p. A mutation in this gene has been associated with amyloid angiopathy by reducing the expression of cystatin C. The frequency distribution of AA, AB, BB (with A as the normal allele and B as the mutated allele) on the CST3 gene in patients of OSMF and healthy counterparts has been probed. The high OSMF risk allele seems to be AA in both low and high exposure groups [40]. However, special attention should be paid to the reduced levels of CST3 when OSMF transforms into malignancy. Further research is required to identify the individual mechanisms operating at various stages and progression to carcinogenesis.

Based on Chiu’s study [40] and other studies, the genotypes associated with the highest OSMF risk in the lower-exposure group were CC of COL1A1, AA of COL1A2, TT of collagenase-1, CC of TGF-β1, AA of LOX and AA of CST3. On the other hand, TT of COL1A1, BB of COL1A2, AA of collagenase-1, CC of TGF-β1, GG of LOX and AA of CST3 genes led to the highest risk to OSMF in the high-exposure group. There was a consistent relationship between genotype distribution of TGF-β1 and CST3 genes and the risk of OSMF in both low- and high-exposure groups, while the other four genes showed inconsistency. The authors further evaluated the effects of the combination of these six genes to examine gene-gene interaction. An elevated risk of OSMF with increasing number of high-risk alleles has been discussed in both high and low exposures for BQ.

## 8. BQ and Plasminogen Activator Inhibitor-1 (PAI-1)

PAI-1 regulates ECM homeostasis and wound healing by suppression of urokinase plasminogen activator (uPA)/tissue plasminogen activator (tPA)-mediated conversion of plasminogen to plasmin, that activates MMPs and fibrinolysis [119]. A number of reports using fibrosis models of internal organs (liver, lung, and kidney) have found that PAI-1 deficiency or inhibition of PAI-1 activity attenuates organ fibrosis [119]. TGF-β may stimulate PAI-1 expression via ROS and smad-dependent (ALK5/smad2/3) and smad-independent (Src/EGFR/MEK/ERK) pathways [120,121]. Interestingly PAI-1 and tPA secretion are increased in fibroblasts derived from OSMF when compared to normal buccal fibroblasts. The ratio of PAI-1/tPA is also increased in OSMF fibroblasts. Arecoline was shown to stimulate PAI and tPA secretion and also increase PAI-1/tPA ratio in buccal mucosal fibroblasts [122,123]. In addition, hypoxia-inducible factor-1α (HIF-1α) over-expression in fibroblasts, epithelial cells and inflammatory cells was found in OSMF tissues relative to healthy tissues. Hypoxia enhanced the arecoline-induced PAI-1 and ECM production by buccal mucosal fibroblasts [124], leading to clinical OSMF. Furthermore, PAI-1 expression was elevated in OSCC tissues relative to normal tissues, but PAI-1 showed little association with the survival rate of OSCC patients [125].

Little is known about the role of PAI-1 polymorphism in the pathogenesis of OSMF and OSCC. The presence of PAI-1 promoter polymorphism −675 4G/5G and high plasma PAI-1 level were reported to increase the risk of keloid in Chinese Han population [103], as well as the risk of liver cirrhosis, hepatocarcinoma and pulmonary fibrosis [126,127]. Recently PAI-1 −675 4G/5G genotypes were found to be strongly associated with overall stage and early stage of OSCC relative to control subjects in the European population [128,129]. More studies are needed to clarify the role of PAI-1 polymorphism in the prediction of risk and survival of OSMF and OSCC patients.

## 9. BQ and TIMPs

There are four TIMPs (TIMP-1, TIMP-2, TIMP-3 and TIMP-4) that show differential inhibitory effects on MMPs, a disintegrin and metalloproteinases (ADAMs), thus preventing ECM proteolysis and leading to accumulation of ECM/tissue fibrosis [56]. Shrestha & Carnelio (2013) found over-expression of MMP-2 and TIMP-2 in mucosal tissues from early and moderately advanced OSMF patients. TIMP-2 is expressed in the lamina propria, deep connective tissue and supra basal layers. MMP-2 is present in the basal and supra basal layers, which showed differences in these two stages of OSMF [130]. Immunohistochemical staining and enzyme zymography analysis found simultaneous increases of MMP-1, MMP-2, MMP-9, TIMP-1 and TIMP-2 expression in OSMF tissues relative to normal tissues [64]. An in vitro study in Sir Lanka also found that fibroblasts from early stage OSMF secrete more TIMP-1 and TIMP-2 than fibroblasts from pan masala chewers with OSMF and normal buccal mucosal fibroblasts. These differences may be caused by replicative lifespan, cellular senescence and premature aging of buccal mucosa tissues [91].

Arecoline and safrole (a component of the *Piper betle* inflorescence) are shown to stimulate TIMP-1 mRNA and protein expression of buccal fibroblasts [73,131]. Fibroblasts from OSMF tissues secreted more TIMP-1 than fibroblasts from adjacent healthy tissues [73,131], suggesting their involvement in the pathogenesis of OSMF. On the contrary, fibroblasts from early stage OSMF showed similar levels in secretion of TGF-β1, MMP-1, MMP-2, interleukin-6 (IL-6), IL-8 and MMP-3 compared to fibroblasts from healthy tissues, but increased TIMP-1 and TIMP-2 secretion of OSMF fibroblasts were noticed [91].

## 10. Conclusions

**Figure 3:**
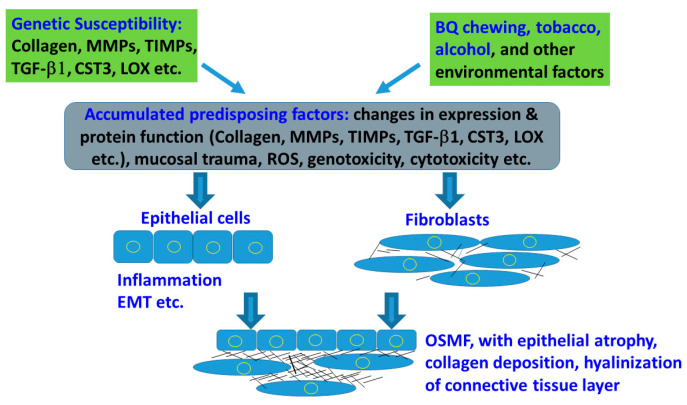
Interactions of genetic factor and environmental factors in mediating the susceptibility and disease processes of OSMF. Factors (Collagen I, MMPs, TIMPs, TGF-β1, CST3 and LOX polymorphisms etc.) and environmental factors (BQ, tobacco, alcohol etc.), mucosal trauma, ROS production, genotoxicity and cytotoxicity may accumulate the predisposing factors, leading to epithelial and fibroblastic changes. The net effect results in OSMF with epithelial atrophy, collagen deposition and connective tissue hyalinization.

OSMF is an OPMD with a potential for malignant transformation. The pathophysiology of OSMF is very complex. The predisposing and risk factors of OSMF and malignant transformation in BQ chewers may vary depending on exposure periods, amount of BQ consumption, with/without other oral habits [tobacco, alcohol], and genetic susceptibility (nucleotide polymorphism of collagen, MMPs, TIMPs, TGF-β1, CST3, and LOX etc.). BQ components including trauma by coarse fiber in AN, catechin, copper, alkaloids, ROS, inflammation, genotoxicity and cytotoxicity are shown as the major contributing factors. These toxic components may stimulate inflammation in the lamina propria of the buccal mucosa, proliferation of fibroblasts and collagen deposition, myofibroblast differentiation and fibrotic contracture, cross-linking of collagen, and may inhibit collagen phagocytosis. The net effect of these events leads to OSMF and oral cancer (Figure 3) but more studies are necessary to fully unravel the underlying molecular mechanisms. BQ components are found to induce extracellular matrix (ECM) deposition via upregulation of TGF-β1, PAI-1, cystatin, LOX, and TIMPs. However, only a small population of BQ chewers develop these diseases, suggesting genetic background plays a role in the development of OSMF. The high risk alleles and genotypes of collagen, MMPs, TGF-β1 and LOX found in OSMF patients with high frequency may change the transcriptional activity and the functions of corresponding proteins, and increase the risk of OSMF. Prevention of mucosal damage, oxidative stress and control of inflammation by stopping of BQ chewing, smoking and alcohol consumption are prerequisites for treatment of OSMF. Surgical management, natural products, low power laser irradiation, enzymes, corticosteroid, vasodilator and antioxidants have been used for treatment of OSMF but their efficacy is limited (Figure 1). New therapies such as targeting therapy toward TGF-β signaling, PAI-1, cystatin or LOX, as well as antioxidative and anti-inflammatory therapy, are urgently needed. It is, therefore, important to further clarify the molecular mechanisms of BQ-induced OSMF and oral cancer in order for future prevention and treatment of BQ-chewing-related diseases.
